# The Role of Plant Latex in Virus Biology

**DOI:** 10.3390/v16010047

**Published:** 2023-12-27

**Authors:** Julia B. Merchán-Gaitán, João H. L. Mendes, Lucas E. C. Nunes, David S. Buss, Silas P. Rodrigues, Patricia M. B. Fernandes

**Affiliations:** 1 Biotechnology Core, Federal University of Espírito Santo, Vitória 29043-900, ES, Brazil; julia.gaitan@edu.ufes.br; 2 Multidisciplinary Core for Research in Biology, Campus Duque de Caxias, Federal University of Rio de Janeiro, Duque de Caxias 25240-005, RJ, Brazil; profjoaoleite.bio@gmail.com (J.H.L.M.); lucasestevao@ufrj.br (L.E.C.N.); 3 School of Life Sciences, Keele University, Newcastle ST5 5BG, UK; d.s.buss@keele.ac.uk

**Keywords:** laticifer, plant-virus interactions, virus, papaya meleira virus

## Abstract

At least 20,000 plant species produce latex, a capacity that appears to have evolved independently on numerous occasions. With a few exceptions, latex is stored under pressure in specialized cells known as laticifers and is exuded upon injury, leading to the assumption that it has a role in securing the plant after mechanical injury. In addition, a defensive effect against insect herbivores and fungal infections has been well established. Latex also appears to have effects on viruses, and laticifers are a hostile environment for virus colonization. Only one example of successful colonization has been reported: papaya meleira virus (PMeV) and papaya meleira virus *2* (PMeV2) in *Carica papaya*. In this review, a summary of studies that support both the pro- and anti-viral effects of plant latex compounds is provided. The latex components represent a promising natural source for the discovery of new pro- and anti-viral molecules in the fields of agriculture and medicine.

## 1. Introduction

Latex is a complex fluid produced by many plants and exuded when plant tissue is damaged through herbivory or physical damage. The color of latex produced by different species can vary from clear to yellow, white, or orange, and the quantity can vary from copious to almost undetectable [1,2]. When exposed to air, it rapidly coagulates, contributing to a reduction in attack by herbivores. This is an advantage for survival in environments with significant populations of herbivorous insects, such as the tropical regions of the planet [1]. The production of latex can vary between even closely related species, with the same species facing different conditions and different parts of the same plant, as shown in *Ficus carica* L. [1].

Latex’s usefulness to plants is evinced by the fact that over 20,000 species produce latex from over 40 families [2], and it has apparently evolved independently on several occasions [2]. Tropical plant species as a group include a higher proportion of latex-producing species than those from temperate regions [3], 14% compared to 6%. However, the purpose of latex was initially unclear. Various theories were put forward over the years, such as storage of nutrients or waste products or maintenance of water balance, but the available evidence offers no support for any of these [2]. For example, although the latex of *Euphorbia esula* contains carbohydrates, these are unavailable to the plant even under conditions of light starvation [4]. The first author to suggest latex as a defense mechanism was James [5], who noted how North American milkweeds produce copious amounts of distasteful latex, which offers “better protection to the plant from enemies than all the thorns, prickles, or hairs that could be provided”. The importance of latex against insect herbivory is now well established, and a role against fungal pathogens has emerged [6].

**Figure 1:**
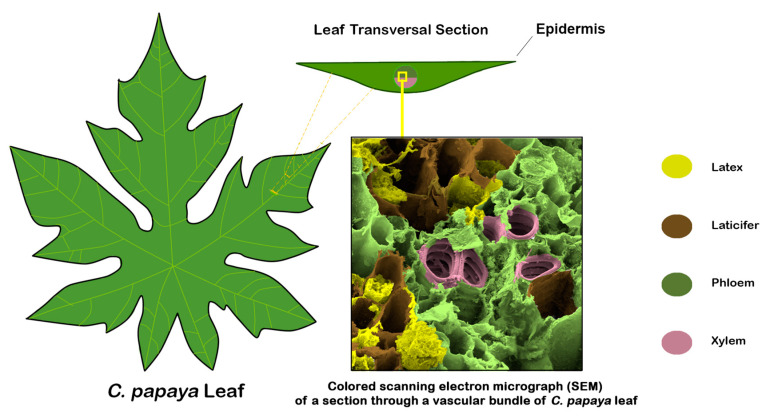
Schematic of the distribution of laticifers in a transversion section of *C. papaya* leaf.

With a few exceptions, latex is produced and stored in specialized cells called laticifers (Figure 1). The convergent evolution of latex production has led to a number of different laticifer structures, although they can be broadly divided into non-articulated and articulated [7]. Non-articulated laticifers originate from cells that elongate and push their way through other cells, branching without cell division but with nuclear division. Neighboring cells do not merge. This group includes the milkweeds (*Asclepias* spp.) and members of the Euphorbiaceae such as *Jatropha dioica*, whose laticifer network is made up of only 5–7 huge cells. Articulated laticifers are formed from chains of cells that merge to form long tubes, and in some species, further merger takes place, forming loops or nets (anastomosing). Examples of articulated laticifers are those found in papaya plants (*C. papaya*), opium poppy (*Papaver somniferum*), and banana (*Musa acuminata*) [7]. 

There is evidence that some plants can at least tailor the contents of their laticifers depending on which part of the plant the laticifer resides in. For example, the white mulberry (*Morus alba*) has non-articulated, branched laticifers that apparently arise independently in different organs of the plant and are discontinuous. In those leaf petioles, two insecticidal chitinases were the most abundant proteins, while in latex from the trunk, an antifungal chitinase was most abundant. These and other variations in content suggest that the laticifers are primed to repel either herbivorous insects from fresh, unlignified leaves or fungal/microbial invaders from the lignified trunk [1]. 

Latexes contain a huge range of secondary metabolites, but the main ones are terpenes, phenolics, alkaloids, and cardenolides. These chemical compounds contribute to environmental plant fitness but not to plant growth itself [1]. Some latex components have an identified, or putative, role in antiviral processes (Table 1). Plant latex also possesses a redox system and a protein-based mechanism that may operate to control virus infection in laticifers. Thus, the aim of this review is to compile information on plant latex’s pro- and anti-viral roles and discuss their potential use. In addition, the application of genetic editing to deepen the understanding of laticifer biology is also presented.

## 2. Protein-Based Mechanisms

### 2.1. Proteases and Their Inhibitors 

*C. papaya* and *Ficus carica* L. (common fig.) latex contain papain and ficin, respectively, which belong to the group of cysteine proteases [22]. The titer of proteases in species of the C1A papain-protease group is very high in leaf tissue and the latex of the fruit, and their participation in the metabolic pathways of different processes such as senescence, abscission, programmed cell death, and fruit ripening has been demonstrated [22]. The extraction and purification of papain from *C. papaya* leaves and fruits have been used in the food industry in winemaking [23], as a meat tenderizer [24], and in the pharmaceutical industry for the production of virus, fungal, and bacterial inhibitor drugs. Also, the use of latex proteases in medicine has been important in providing treatments for degenerative diseases due to their enzymatic action [22]. 

The latex exuded by *C. papaya*, a product of the laticifers that are damaged during the attack of herbivorous insects, has a toxic effect on some caterpillars, such as the cabbage moth (*Mamestra brassicae*) [25]. However, this effect disappears if the latex is washed or a cysteine protease inhibitor is added [26], such as the digestive substances of the monarch caterpillar (*Danaus plexippus*), which effectively destroys the toxicity of the latex [27]. A few prominent plant families that produce a larger amount of latex are the Caricaceae, Moraceae, and Apocynaceae, which also contain cysteine proteases in their latex, while serine proteases [28] have been found in latex samples from the Moraceae, Euphorbiaceae, Apocynaceae, and Convolvulaceae [29]. Several proteases known to be activated in laticifers are involved in the hypersensitivity response in plants to virus infection. However, the direct effect of cysteine proteases on viruses is poorly understood [30,31]. 

When infected by viruses, plants develop a local hypersensitive response (HR) that usually involves programmed cell death (PCD) [31]. Apoptosis, a form of animal PCD, involves cysteine proteases of the caspase group that targets specific protein substrates [32], and in plants, the activity is affected by metacaspases and papain-like Cys proteases that are induced during HR [33]; however, these activities are inhibited when caspase inhibitors are used, but protease inhibitors that do not inhibit caspases, e.g., E-64 and AEBSF, can also block PCD [30,34]. This suggests that additional proteases, such as papain-like cysteine proteases (PLCPs), can also be effectors or regulators of plant PCD [34]. In fact, cathepsin B, a papain-like cysteine protease, was shown to be required for disease resistance-causing HR in plants [35]. Against this hypothesis, the infection of *C. papaya* laticifers by papaya meleira virus (PMeV) and papaya meleira virus 2 (PMeV2) [36] leads to the accumulation of hydrogen peroxide (H_2_O_2_) [17], generating a reduction in the levels and activity of cysteine protease [30]. However, it could be argued that these are, by definition, susceptible plants, and a reduction in protease levels may be associated with their susceptibility [27,35].

There are many examples of cysteine and serine protease inhibitors in plant latex. However, a discernible function of these inhibitors seems to involve the inhibition of insect herbivory and microbial activity, including certain viruses [25]. Thus, a role in anti-viral protection is becoming apparent. Many viruses themselves encode proteases whose proteolytic enzymatic activity could act against host defenses [37]. For this reason, several protease inhibitor drugs, including azithromycin, were tested in the last coronavirus pandemic, demonstrating a clear example of their increasing use in clinical medicine as antiviral agents [38]. Expression of the rice cysteine protease inhibitor gene oryzacystatin I in transgenic tobacco showed enhanced resistance to two potyviruses, tobacco etch virus and potato virus Y, which use cysteine proteases for protein processing [8]. Specific protease inhibitors are also expressed naturally in response to viral infections. Tomato plants infected with tomato spotted wilt virus express an unusual protease inhibitor with some features of cysteine and serine inhibitors, although the effect on the virus is unclear [9].

### 2.2. Loss-of-Susceptibility to Potyviruses (LSP1) Protein 

A proteomic survey of lettuce latex [39] found a putative LSP1 protein, a member of the eukaryotic translation initiation factor 4E (eIF4E) family of proteins, which are involved in translation initiation. The LSP1 protein is an isoform called eIF(iso)4E, and mutation of this gene is one of the factors associated with resistance to potyvirus infection in *Arabidopsis thaliana* [10]. This protein is a cap-binding protein that binds to the 5’ cap structure of nuclear-encoded mRNAs. The translation factor eIF(iso)4E can interact with the potyviral genome-linked protein of some potyviruses, apparently in a pro-viral manner, as *Arabidopsis* mutants lacking eIF(iso)4E have immunity or near immunity to turnip mosaic potyvirus and tobacco etch potyvirus [40]. The LSP1 protein is thought to play a role in the translation of mRNAs that are important for the establishment of potyvirus infection [10]. The identification of eIF(iso)4E in lettuce latex in association with proteins from the lettuce mosaic virus, a potyvirus, implies its potential involvement in susceptibility within this particular species [39]. 

### 2.3. Ubiquitin-Proteasome Degradation

Various proteins associated with the ubiquitin proteasome-mediated protein degradation system have been detected in lettuce latex, including a putative CAND1 (Cullin Associated and Neddylation Dissociated 1), an unmodified CUL1-interacting protein [41], More Axillary Branch 2 (MAX2) (a type of ubiquitin-protein ligase), and the 20S proteasome beta subunit G1 [42]. Similarly, expression of ubiquitin–proteasome pathway genes has been detected in latex from the rubber tree (*Hevea brasiliensis*) and fig fruit (*Ficus carica* L.) [43,44].

Upregulation of genes associated with the ubiquitin–proteasome system in response to virus infection and propagation has been reported by a number of studies, for example in *Arabidopsis* following plum pox virus infection [45] or tobacco (*Nicotiana tabacum*) infected with tomato mosaic virus or tobacco mosaic virus [46]. Conversely, silencing of the E3 ubiquitin ligase NbSGT1, which operates as a co-chaperone during viral infection, and plant immunity generated by R genes in *Nicotiana benthamiana* compromise resistance to tobacco mosaic virus [47] and potato virus X [48,49], though not cauliflower mosaic virus [48]. Proteomic analysis of *C. papaya* leaf samples showed that both 20S catalytic and RPT5a subunits were upregulated following PMeV infection [18]. Those proteins were found later to occur in the *C. papaya* latex proteome, although their abundances did not change significantly during infection [30]. However, the ubiquitin–proteasome system (UPS) may function in a dual role during virus infection by impairing or facilitating viral replication or transport. This is part of the general resource redistribution within the plant body due to infection or a direct down-regulation effect on viral proteins or proteins essential for virus replication, as has been reported for various virus movement proteins. There may even be an effect on the virus’s RNA or DNA integrity, as the sunflower proteasome has been shown to act as a nuclease on tobacco mosaic virus in vitro [49].

As with most aspects of plant-pathogen relationships, there are pathogen responses to plant defenses. Lettuce mosaic virus infection leads to an inhibition of proteasome RNase activity in pea plants, apparently by binding the viral protein helper component-proteinase (HcPro) to multiple sites on the 20S proteasome core [50,51].

### 2.4. Heat Shock Protein 70 (Hsp70) Isoforms

The plant Hsp70 protein chaperone system has been previously shown to be co-opted by infecting viruses. Hsp70 family proteins have been associated with the assembly of viral replicases, stimulation of viral RNA-dependent RNA polymerase (RdRp) activity, intracellular transport of replication proteins, and generally the assembly and activity of viral proteins [19]. Infected plants typically express elevated Hsp70 levels [52], while silencing or inhibition of Hsp70 reduces tomato bushy stunt virus [53] accumulation. Contrarily, infection of *C. papaya* by PMeV and PMeV2 downregulates Hsp70 proteins in leaf tissue [18]. Several Hsp70 isoforms have been found in the *C. papaya* latex proteome. Although not statistically significant, almost all of them were in lower abundance in the infected latex [30]. This suggests that *C. papaya* Hsp70s are also important for PMeV/PMeV2 replication, and lowering the abundance of those proteins may limit viral replication. 

## 3. Oxidative Responses

Production of reactive oxygen species (ROS) such as H_2_O_2_ and nitric oxide (NO) is a well-established signaling mechanism and is also involved in response to viral infection [54]. *C. papaya* laticifers infected with PMeV and PMeV2 show considerable increases in H_2_O_2_, phosphorus, potassium, and water levels that cause the spontaneous exudation of latex in fruits, perhaps due to an osmotic alteration [17,30]. In addition, a reduction in calcium together with an increase in hydrogen peroxide might affect signaling in the regulation of laticifer stress. In accordance with this, several isoforms of peroxidase have been isolated from *C. papaya* latex [30]. Similarly, the presence of antioxidant enzymes involved in plant defense has been demonstrated in the latex of lettuce (*Lactuca sativa*) and yellow bellflower (*Thevetia peruviana*) [55]. They include methionine sulfoxide reductase, which can rescue enzymatic activity after damage to methionine residues by ROS such as hydroxyl radicals and superoxide ions [56]. Three enzymes involved in thioredoxin reduction and oxidation, and thus cellular redox balance, were also isolated: ferredoxin oxidoreductase, ferredoxin thioredoxin reductase, and mitochondrial NADP adrenodoxin-like ferredoxin reductase [56]. Antioxidant production is one area that illustrates the disparate evolution of lactifers, as shown by a survey of three plant species from Brazil [27,57]. Latex from *Cryptostegia grandiflora* (Apocynaceae) and *Plumeria rubra* (also Apocynaceae) exhibited strong ascorbate peroxidase and superoxide dismutase activities [58], while activity in *Euphorbia tirucalli* (Euphorbiaceae) latex was undetectable [59]. In contrast, only *C. grandiflora* exhibited measureable catalase activity [60].

## 4. Secondary Metabolites

### 4.1. Phenolics and Polyphenols 

Phenolic compounds are secondary metabolites involved with biotic and abiotic stresses in plants [61]. The chemical complexity of these compounds ranges from simple phenolic acids to complex tannins and lignins. Polyphenols have structural diversity because of the number of their phenol rings and other linked elements. They are present in several parts of plants (leaves, fruits, roots, etc.), including latex [61].

*Hevea brasiliensis* latex has quercetin and rutin, both polyphenols with relevant antiviral activity against rabies virus, poliovirus, syncytial virus, HSV-2, respiratory syncytial virus, dengue virus, influenza virus, and coronavirus [62]. The antiviral mechanism of these compounds is related to inhibition of viral polymerase and binding of viral nucleic acid [63]. 

SP-303/Provir, an oligomeric proanthocyanidin polyphenol isolated from the latex of *Croton lechleri*, has been shown to have an antiviral action against a number of human viruses, including respiratory syncytial virus, influenza A virus, parainfluenza virus, and herpes virus types 1 and 2. SP-303 seems to bind directly to the viral envelope, inhibiting viral attachment [11]. 

Some plants contain large amounts of phenolics in their latex; for instance, the sweet potato, *Ipomoea batatas*, contains the hexadecyl, octadecyl, and eicosyl esters of p-coumaric acid in up to 10% of fresh root latex in one variety [64]. However, tests of p-coumaric acid with herpes simplex virus type 1 [65] and type 2 [65] or human cytomegalovirus [66] found no antiviral effect, although there was a degree of antiviral activity against one of the adenoviruses tested [65].

### 4.2. Terpenoids 

Terpenoids are one of the most prevalent secondary metabolites in plant latex [67] and consist of isoprene subunits. They present promising antimicrobial activity against bacteria, fungi, protozoa, and viruses. The latex of lettuce is especially rich in sesquiterpene lactones, including lactucin, lactucopicrin, and lettucenin A [68], which have been shown to deter insect feeding as well as have an antifungal action, inhibiting the growth of *Cladosporium herbarum* [68]. Although there have been suggestions that latex terpenoids contribute to the resistance of *Lactuca* species to multiple viruses, a comparison of sesquiterpene lactone titers and resistance levels suggests that this is not the case [68]. 

### 4.3. Cardenolides 

Cardenolides, a type of steroid, are found in the latex of various Apocynaceae plants, including milkweeds (*Asclepias* spp.) and oleander (*Nerium oleander*), as well as the latex of a Moraceae species, *Antiaris toxicaria*, apparently arising from convergent evolution [69]. A cardenolide derivative, glucoevatromonoside, isolated from a Brazilian cultivar of the Woolly foxglove (*Digitalis lanata*), has been shown to inhibit replication of herpes simplex virus types 1 and 2 [12]. It apparently inhibits viral protein synthesis as well as reduces cellular release and spread of virus, likely as an indirect result of virus-caused inhibition of Na+K+ATPase activity. It thus leads to cellular depletion of K^+^ and lowers the activation of several viral K^+^ dependent enzymes.

### 4.4. Alkaloids 

Alkaloids are a group of compounds found in plants, animals, and fungi derived from amino acids [1]. In plants, alkaloids are found as non-volatile and non-odorous compounds in different tissues, including laticifers. One of the most known alkaloids is opium, obtained from the latex of *P. somniferum* and commonly used in medicine and psychedelic drugs [1]. In addition to the classical anti-herbivorous activity [67], some alkaloids affect virus replication. For example, ChM-P2 from *Chelidonium majus* latex prevented infection with HIV-1 in vitro and in vivo. Moreover, patients infected with SARS-CoV-2 treated with *C. majus* latex showed clinical improvement after three days of treatment [1]. The mechanisms involved in the antiviral activity of alkaloids include DNA and RNA synthesis inhibition and viral replication blockage [70]. Several studies showed that alkaloids interact with cell membrane receptors, indirectly disturbing the cell-virus interaction. This might have an effect on several viruses, as enveloped and non-encased viruses rely on the cell membrane as the main site for cell cycle completion. Alkaloids may have other targets; for instance, oliverine suppresses HSV-1 DNA synthesis, lycorine inhibits dengue virus and zika virus RNA polymerases, emetine inhibits HIV-1 reverse transcriptase, and tomatidine interferes with the production of CHIKV viral particles [70]. Therefore, plant laticifers are important sites for bioactive compound production and storage [67].

## 5. Trials of the Biological Activity of Latex Constituents

### 5.1. Antiviral Activity

A number of trials have found fractions or compounds from latex to have an effect on viruses. As early as 1974, it was found that latex from three *Jatropha* sp. (Euphorbiaceae) inhibited infection with the tobacco mosaic virus. Other studies have been influenced by traditional medicine; for example, latex from the fig *Ficus carica* has been used to treat diseases supposedly caused by viruses. Therefore, it was tested for antiviral activity against herpes simplex type 1 (HSV-1), echovirus type 11 (ECV-11), and an adenovirus [13]. Hexane and hexane-ethyl acetate fractions were found to inhibit virus multiplication at levels below cell toxicity. Studies are often based on members of the Euphorbiaceae used in traditional medicine. It was found that a methanol extract of latex from *Codiaeum variegatum* is active against the influenza virus A/PR/8/34, while a triterpene extract of *Euphorbia tirucalli* latex is active against rhinoviruses [14].

The hunt for compounds with anti-HIV activity has also uncovered antiviral compounds from plant latex. A survey of trees in Malaysia yielded the dipyranocoumarin calanolide B, isolated from the latex of *Calophyllum teysmanii*, which went through preclinical trials [15]. Calanolide A and B act by inhibiting HIV-1 type 1 reverse transcriptase and have since been found in the bark and leaves of various *Calophyllum* species [15]. 

A study with *Chelidonium majus* L. (Papaveraceae) latex showed that compounds of latex, such as alkaloids and proteins, decreased HPV infection and inhibited oncogene viral expression [71]. *C. majus* has been used in Asian and European natural treatments of condylomas, which are visible HPV symptoms [71]. 

A glucoside lignin ((+)-pinoresinol 4-*O*-[6″-*O*-vanilloyl]-*β*-d-glucopyranoside) was isolated from the latex of *Calotropis gigantea* (Asclepiadaceae) and showed antiviral activity against influenza (H1N1) through suppression of viral replication [72]. Other compounds (6′-*O*-vanilloyltachioside and 6′-*O*-vanilloylisotachioside) were found but did not present anti-influenza activity [72]. 

A virus-cell-based assay with chikungunya virus (CHIKV) subjected to *Euphorbia* latex showed more potent antiviral activity than other extracts tested. 13-O-isobutyryl-12-deoxyphorbol-20-acetate and ingenol-3-mebutate were found in the latex of *Euphorbia* species (*E. peplus* and *E. segetalis ssp. pinea*) and can be related to inhibition of the CHIKV [73].

### 5.2. Proviral Activity

Perhaps surprisingly, there have been a number of studies showing activation rather than suppression of viral activity. An example comes from endemic Burkitt’s Lymphoma (eBL), one of the commonest childhood cancers in sub-Saharan Africa, which is closely associated with the Epstein–Barr virus (EBV). Comparison of the distribution of the spurge *Euphorbia tirucalli* with that of eBL [74] and the discovery that methanol extracts of *E. tirucalli* tissues enhanced EBV-mediated cell transformation [75] led to suggestions that this plant may be involved as a cofactor in eBL, though it was initially unclear how. Like many of the Euphorbiaceae, *E. tirucalli* exudes a milky white latex that can be used as glue and is commonly played with by children. It was found that this latex applied to cell lines activated the EBV lytic cell cycle, implicating *E. tirucalli* latex as a possible environmental co-factor in eBL [20]. One suggestion is that 4-deoxyphorbol esters in the latex act in conjunction with EBV to alter c-MYC expression and cause chromosome aberrations [76]. 

Virus reactivation can be clinically useful. A major obstacle to HIV-1 eradication with antiviral mixtures (Highly Active Antiretroviral Therapy, or HAART) is the presence of latent HIV-1 cell reservoirs, which typically reactivate when HAART treatment is interrupted [77]. Thus, concurrent virus activation and HAART treatment are desirable options. Various plant products have proven useful in this regard, such as prostratin [78], and studies suggest that terpenoids isolated from the latex of *Euphorbia lactea* and *E. laurifolia* [79] or ingol diterpenes from the latex of *E. officinarum* [21] can also cause HIV-1 activation. The compound from *E. lacteal* likely acts via the PKC pathway [79].

## 6. Papaya Meleira Virus Complex: Two Viruses Infecting Laticifers

Although a proteomic analysis of lettuce latex found evidence of proteins from lettuce mosaic virus, mirafiori lettuce big-vein virus, lettuce big-vein virus, lettuce infectious yellows virus, lettuce ring necrosis virus, and lettuce necrotic yellows virus [39], it is remarkable that, so far, only five viruses, PMeV, PMeV2, a Mexican variant of PMeV (PMeV-Mx), papaya virus Q (PpVQ), and papaya sticky fruit associated virus (PSFaV), have been isolated from latex samples [80]. This suggests that viruses have their transmission to plants impaired by the physical and chemical barrier formed by latex coagulation upon insect feeding on plant tissues [69]. Alternatively, latex is either a hostile environment for viruses or this plant fluid has been neglected in the plant virus field. 

PMeV is an 8.7 kbp double-stranded RNA (dsRNA) virus (*Fusagraviridae*) [81] that, on co-infection with PMeV2, a 4.5 kbp single-stranded RNA (ssRNA) (umbra-like virus) [36], causes papaya sticky disease (PSD) [36,82]. Sequence similarity and phylogenetic analysis that included PMeV2, PMeV-Mx, and PpVQ suggest that these viruses may be different isolates from the same umbravirus [36]. Thus, PSD symptoms are caused by the combined infection of PMeV and PMeV2 (the PMeV complex) [80]. 

There are two fundamental questions to be answered: how can the PMeV complex colonize *C. papaya* laticifers, and what is its dispersion strategy within the plant and in the environment. The first is as yet unclear, but there has been progress with the second, and changes within the latex following infection have been determined [17,30]. 

The latex from diseased plants coagulates much less readily than that from healthy plants, and latex particles have a different morphology and a reduced titre. Viral particles bind tightly to the latex solid phase, which appears to change its morphology. The model of latex coagulation from the rubber tree results from the binding of surface proteins on the latex particles, specifically Hevea latex lectin-like protein (HLL) to glycosilated N-Acetyl-D-glucosamin (GluNAC) receptors, causing particle aggregation [83]. Thus, changes in surface morphology in *C. papaya* latex may disrupt coagulation, resulting in a more liquid latex. There are also biochemical changes within the latex. Binding between HLL and GluNAC in rubber trees is calcium-dependent [83]. The latex from sticky diseased *C. papaya* contains about half the amount of calcium in the latex of healthy plants [17], although there is an increased concentration of insoluble calcium oxalate.

*C. papaya* latex infected by the PMeV complex also contains about half the sugar concentration of latex from asymptomatic plants. Reduction of plasmodesmata diameter in plants has been observed in response to viral infection, presumably as a means to reduce viral mobility, and this can restrict the transport of sugars from photosynthetic sugars. *C. papaya* latex consists primarily of proteins and polyisoprene molecules, and a reduction in carbohydrates would be expected to reduce the biosynthesis of both, similar to the effect on isoprene synthesis in *Hevea* [84]. PMeV complex-infected *C. papaya* latex also shows an increased level of potassium, possibly due to the inward rectifying K^+^ channel in the laticifers. This would be expected to increase water intake and thus could account for the spontaneous bursting of laticifers and exudation of latex seen in infected tissues. Taken together, changes in the latex particles’ shape, interference with their binding, and an increased water content could account for the main symptoms of PSD. 

But how does the PMeV complex deal with the hostile latex environment? There does appear to be a plant defense response; for example, alkaloids were elevated in infected latex [17], and these have been shown to be effective against viruses in other circumstances. Infected laticifers have an elevated level of H_2_O_2_ production, which is a known signaling mechanism for viral infection, and it has been suggested that the same is occurring here, especially as production is localized adjacent to phloem cells, which have been correlated with systemic transport and the stress response [17]. Further downstream in the resistance gene regulatory pathway, the Ran gene [85] and Ran/TC4 are upregulated during PMeV complex infection [18]. Further indication of a defense response comes from the upregulation of calreticulin [18], a calcium-binding protein. Calreticulin expression has been reported as a response to viral infection in other plant species and is an essential component of the plant Ca^2+^ signaling system, which is known to activate resistance pathways [86]. In addition, calreticulin has been shown to bind directly to the tobacco mosaic virus, inhibiting its spread through the tobacco host [16], and in *C. papaya*, it binds to the helper component proteinase of the papaya ringspot virus (PRSV) [16]. Similarly, the downregulation of *C. papaya* translation initiation factor, Hsp70, and glyceraldehyde 3-phosphate dehydrogenase (GAPDH) [18] may be a plant response to limit viral replication.

Although these responses of *C. papaya* to PMeV complex infection may account for the absence of viral infection outside of laticifers, the success of the virus in colonizing laticifers is still unexplained. As we have seen, plant latex is a highly hostile environment, with high concentrations of proteases and other anti-pathogenic compounds. An equivalent is perhaps the insect midgut, again rich in protease enzymes, and an example from there gives an idea of the capacity of viruses to survive harsh environments [87]. Cytoplasmic polyhedrosis virus (CPV) is a dsRNA virus enclosed in a capsid shell made up of 120 molecules of inner capsid shell protein. Virions of CPV are resistant to chemical treatments such as high pH and SDS and to enzyme disruption by trypsin, chymotrypsin, ribonuclease A, deoxyribonuclease, and phospholipase [88]. It is also capable of endogenous mRNA transcription within an intact virus particle using viral-encoded enzymes [87]. Although PMeV complex particles have been isolated and their genome and structural proteins have been studied [81], how *C. papaya* latex components influence PMeV/PMeV2 replication and effects on the plant’s laticifers remains largely unknown. 

The PMeV complex is able to colonize *C. papaya* laticifers, is able to move within the plant, and is able to infect other plants. There has been little study of cytoplasmic streaming or long-distance transport within laticifers (reviewed in [89]), but transport from shoots to roots via laticifers in cassava has been proposed [90]. Such movement would provide a transport mechanism for the PMeV complex and any other laticifer viruses in the plant. Additionally, of course, rupture of a laticifer causes movement of latex within the laticifer, and this would also transport the virus, both within the laticifer and outside, to the plant body. It has been proposed that the characteristic symptom of PMeV complex infection, spontaneous exudation of watery, sticky latex, is a strategy of the virus to aid dispersion [18]. 

## 7. Plant Genetic Editing of Laticifer’s Expressed Genes 

Genetic editing of plants, mostly by CRISPR (clustered regularly interspaced short palindromic repeats), has been used to alter plant genes involved in disease control, stress, and the functional analysis of proteins [91]. As such, some genes typically expressed in laticifers have been edited (Table 2) to understand laticifers’ biology and to control latex composition. 

The transcriptome analysis of latex mRNA from different plant species, e.g., *Hevea brasiliensis* Muell., *Ficus carica* L., *Taraxacum kok-saghyz*, *Papaver somniferum*, and *Euphorbia tirucalli*, has revealed laticifer active promoter regions. In parallel, a *H. brasiliensis* U6 mutant promoter induced by ribonucleoproteins has been identified and characterized. The mutation frequencies ranged from 3.74% to 20.11% among the five targeted sites [99]. Five genes of the HbFT family involved with flowering control were edited by CRISPR/Cas9. Thus, their results point to flowerless plants with increased latex production and a reduced energy loss phenotype [98]. 

*Taraxacum koksaghyz* is known as the rubber dandelion and produces high-quality, high-molecular-weight rubber particles. Several studies [92,94] have used CRISPR/Cas9-based methods to understand rubber biosynthesis and to accelerate *T. koksaghyz* domestication to be used as a rubber-producing crop. By inducing mutagenesis at the fructan 1-fructosyltransferase (1-FFT) encoding gene, mutation rates ranging from 39.4% to 88.9% were obtained. The edited plants showed shorter flowering times than wild-type plants and flowered at the sixth week [94,96]. The 1-FFT enzyme is involved in the biosynthesis of inulin, a storage carbohydrate. Similarly, overexpression of fructan-1-exohydrolase, which degrades inulin, leads to an increase in *T. koksaghyz* natural rubber synthesis [96]. 

The role of the *T. koksaghyz* rapid alkalinization factor-like 1 gene (TkRALFL1) in inulin and rubber production has also been evaluated using CRISPR-based gene editing [92]. Thus, heterozygous and homozygous TkRALFL1 knockout plants showed a slight increase in inulin and rubber content compared to control plants [92]. In the same species, the PEP16 gene promoter from *H. brasiliensis* (HbPEP16), which has cis-acting elements responsive to several plant hormones and is involved with rubber biosynthesis, was tested. In lettuce, CRISPR/Cas9 targeting the laticifer-specific cis-prenyltransferase 3 was used to generate rubber production-deficient mutants. The edited plants showed a considerable decrease in rubber production. They were further genetically modified through the introduction of the guayule and goldenrod genes, which are involved with rubber production and rubber polymers’ properties. The latex from those plants showed rubber composed of longer polymers than those of control plants [93]. 

A gene editing study was also carried out in chicory (*Cichorium intybus*) to inactivate the kauniolide synthase genes (*Cikls1*, *Cikls2*, and *Cikls3*), classified in the CYP71 family of cytochrome P450 enzymes, and analyze the resulting effects on the production of sesquiterpene lactones (STLs). The roots of edited plants showed an accumulation of costunolide and its conjugates. Although pivotal roots of edited *C. intybus* lines have shown increased free costunolide content, the major STLs and STLs-derived oxalates were completely absent in some lines compared to controls [97]. The same species had the germacrene synthase gene (*cigas*) edited. As *cigas* are involved with STL biosynthesis, the edited plants showed a strong reduction in STL levels and an accumulation of phenols and squalene [95]. The authors highlighted the potential use of those genetic lines for improved inulin extraction. 

Although there are no publications reporting expressed genes in laticifers whose products interfere with virus infection as gene editing targets, the field is expected to grow in the next few years as the protocols for genetic editing of the above-cited species have been tested and are available for further studies.

## 8. Conclusions

Laticifers have independently evolved in various plant species, and numerous studies indicate their specialization as plant defensive structures. Latex exudation serves to seal wounds, acting as a deterrent and potentially lethal agent for herbivorous pests. Increasing evidence suggests that latex may also play a role in plant responses to viral infections, given the observed antiviral properties of many latex components and the relative absence of virus colonization in laticifers, albeit not entirely devoid. This area of research is expanding, particularly with the advent of gene-editing tools, offering novel insights into both plant biology and medicine.

## Figures and Tables

**Table 1:** Confirmed effects of plant laticifer components on viruses and potential interactions inferred from other plant tissues.

ANTIVIRAL INTERACTION			
Component	Interaction	Model	Reference
alkaloids	DNA and RNA synthesis inhibition and viral replication blockage	HIV-1, SARS-CoV-2	[1]
cysteine protease inhibitor	inhibition of viral cysteine proteases	potato virus Y in potato plants	[8]
cysteine/serine protease inhibitor	inhibition of viral proteases	tomato-spotted wilt virus in tomato	[9]
ubiquitin–proteasome system	degradation of viral proteins	potyvirus in *Arabidopsis thaliana*	[10]
phenolics and polyphenols	binding to the viral envelope	influenza A virus in *Croton lechleri*	[11]
cardenolides	inhibition of viral protein synthesis, viral cellular release, and dispersion	herpes simplex virus type 1 in *Digitalis lanata*	[12]
latex extracts	inhibition of viral multiplication	herpes simplex virus type 1 and echovirus type 11 in *Ficus carica*	[13,14]
calanolides	inhibition of viral reverse transcriptase	*Calophyllum teysmanii*, a human immunodeficiency virus	[15]
calreticulin	binding to the virus to inhibit spread or to the virus helper protein	tobacco mosaic virus in *Nicotiana tabacum*	[16]
**PRO-VIRAL INTERACTIONS**			
**Component**	**Interaction**	**Model**	**Reference**
latex particles	potential increase in latex fluidity, increasing viral dispersal	papaya meleira virus in *Carica papaya* L.	[17]
Hsp70 proteins	assembly and activation of viral proteins, reduction in plants in response to infection	tomato bushy stunt virus in *Nicotiana benthamiana* and *Carica papaya* L.	[18,19]
*Euphorbia tirucalli* latex	activation of the Epstein-Barr virus lytic cell cycle	Epstein-Barr virus in *Euphorbia tirucalli*	[20]
terpenoids	activation of the HIV-1 virus	human immunodeficiency virus type 1 in *Euphorbia officinarum*	[21]

**Table 2:** Genes typically expressed in laticifers are edited using CRISPR.

Gene	Function	Plant Host	Reference
rapid alkalinisation factor like 1	influences root phenotype and biomass, and inulin and natural rubber yield	*Taraxacum koksaghyz*	[92]
laticifer-specific cis-prenyltransferase 3	involved in high-quality rubber production by laticifers	*Lactuca sativa*	[93]
1-fructosyltransferase	encodes a key enzyme in inulin biosynthesis	*Taraxacum koksaghyz*	[94]
germacrene A synthase	involved with the degradation of sesquiterpene lactones	*Cichorium intybus* L.	[95]
rubber elongation factor and small rubber particle protein	belong to the stress-related protein superfamily involved in rubber biosynthesis and storage	*Taraxacum koksaghyz*	[96]
kauniolide synthase	disruption of sesquiterpene lactone biosynthesis in laticifers	*Cichorium intybus var. sativum*	[97]
ribonucleoprotein	belong to a family that controls plant flowering time	*Hevea brasiliensis*	[98]

## Data Availability

No new data were created or analyzed in this study. Data sharing is not applicable to this article.
